# The anti‑inflammatory activity of 2‑iminothiazolidines: the role of PPARγ and M2 macrophage subpopulations

**DOI:** 10.1007/s10787-026-02253-y

**Published:** 2026-04-22

**Authors:** Eduarda Talita Bramorski Mohr, Tainá Larissa Lubschinski, Pedro Augusto Marocco Moro, Larissa Benvenutti, Natáli Tereza Capistrano Costa, Pedro Luís Pereira Braga de Sousa, Marcus Mandolesi Sá, Marcelo Zaldini Hernandes, Flora Aparecida Milton, José Roberto Santin, Eduardo Monguilhott Dalmarco

**Affiliations:** 1 Department of Clinical Analysis, Center of Health Sciences, Federal University of Santa Catarina (UFSC), Campus Universitario‑Trindade, Florianopolis, SC 88040‑900 Brazil; 2 Department of Chemistry, Federal University of Santa Catarina, Campus Universitario‑Trindade, Florianopolis, SC 88040‑900 Brazil; 3 Postgraduate Program in Pharmaceutical Sciences, Universidade Do Vale Do Itajaí (UNIVALI), Itajaí, SC 88302-901 Brazil; 4 Laboratory of Medicinal Theoretical Chemistry (LQTM), Department of Pharmaceutical Sciences, Federal University of Pernambuco (UFPE), Recife, PE 50740-521 Brazil; 5 Laboratory of Molecular Pharmacology, School of Health Sciences, University of Brasilia (UnB), Brasilia, DF 70910-900 Brazil

**Keywords:** Thiazolidines, PPARγ agonists, NF-kappa B, Macrophage activation, Macrophage-activating factors

## Abstract

Thiazolidine derivatives exert anti-inflammatory effects by inhibiting the NF-κB pathway and promoting macrophage polarization toward the anti-inflammatory M2 phenotype, partially through PPARγ activation. Our previous work identified thiazolidine derivative **13** [methyl 2-(benzoylimino)-3-methyl-4-(4-nitrobenzyl)-1,3-thiazolidine-4-carboxylate] as a potent immunomodulator that reduces pro-inflammatory mediators and enhances anti-inflammatory markers. However, its direct interaction with PPARγ remains unclear. This study aims to evaluate whether thiazolidine **13** activates PPARγ and contributes to M2 macrophage polarization using LPS-stimulated RAW 264.7 cells, cell transactivation assay and reporter gene (Hella cells), LPS-stimulated THP-1 cells, and molecular docking analysis. **Compound 13** reduced NO₂⁻ production, CD80 expression, and pro-inflammatory cytokines while increasing IL-10, TGF-β, and CD206 in LPS-stimulated RAW 264.7 macrophages. **Compound 13** also inhibited NO production in LPS-stimulated THP-1 cells. These effects were only partially reversed by PPARγ antagonists, suggesting additional PPARγ-independent mechanisms, while the induction of CD206 was closely linked to partial PPARγ activation. Docking studies supported this profile, showing that the (*S*) enantiomer of thiazolidine **13** adopts a binding mode typical of partial PPARγ agonists and exhibits a higher predicted affinity than the (*R*) enantiomer. **Compound 13** acts as a partial PPARγ modulator capable of shifting macrophages from a pro-inflammatory toward an anti-inflammatory phenotype (M2-like). This dual action relies on the inhibition of NF-κB signaling and partial PPARγ activation. The predominance of the M2-like subpopulation, underscores its role in resolving inflammation and promoting tissue repair.

**Graphical abstract:**

**Figure:**
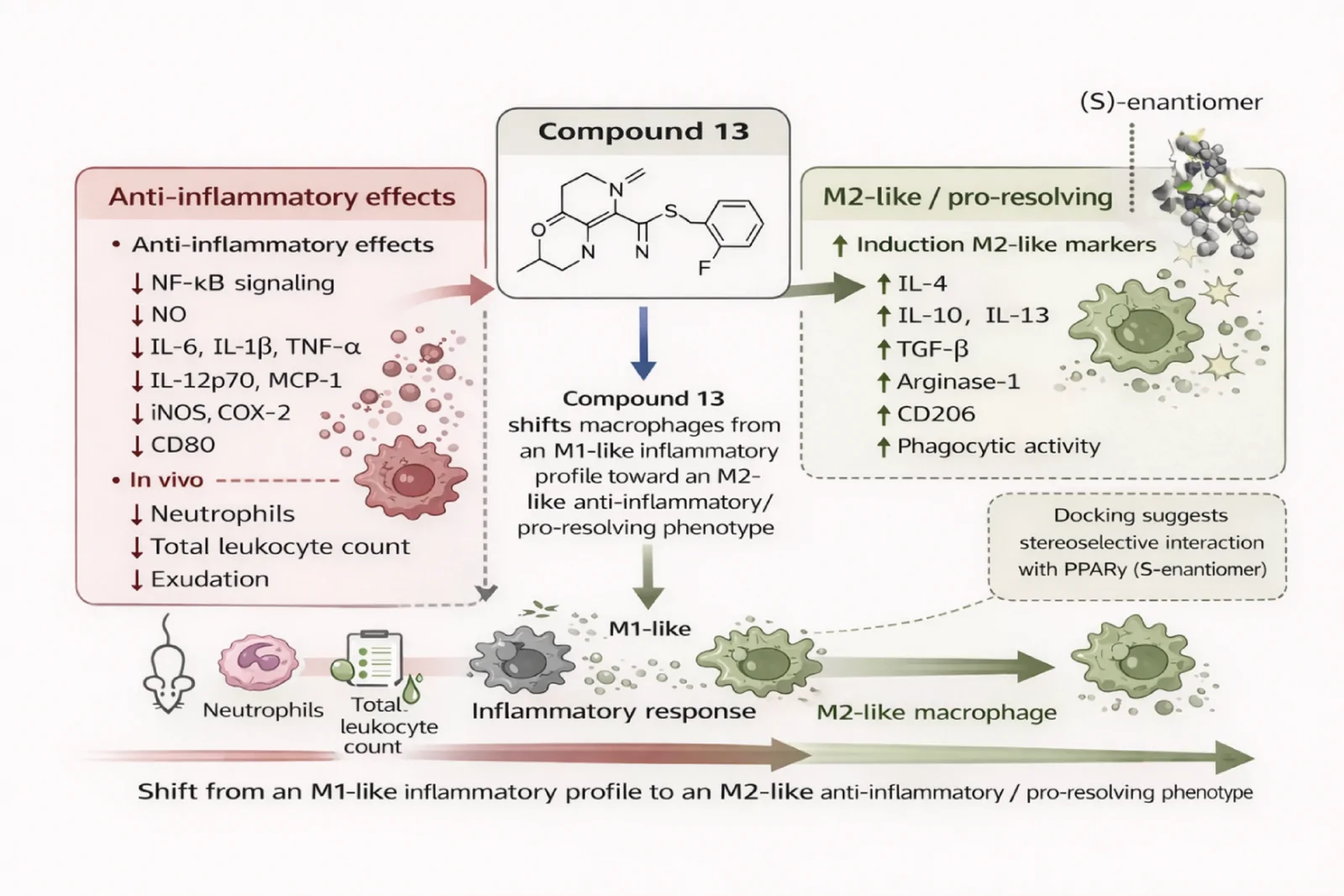

**Supplementary Information:**

The online version contains supplementary material available at https://doi.org/10.1007/s10787-026-02253-y.

## Introduction

Thiazolidine derivatives exhibit various biological activities, with their heterocyclic ring structure serving as a foundation for developing new pharmacological compounds (Coin et al. 2021). Notably, these molecules have anti-inflammatory effects, reducing markers such as nitric oxide (NO), prostaglandin E2 (PGE_2_), and cytokines, such as Tumor necrosis factor- α and Interleukine-6 (TNF-α and IL-6) (Havrylyuk et al. 2016; Mohr et al. 2025). Moreover, the effects are closely linked to inhibition of the NF-κB pathway, which plays a key role in regulating inducible nitric oxide synthase (iNOS) and cyclooxygenase-2 (COX-2), enzymes essential for initiating and sustaining inflammatory processes (Bhatnagar & Pemawat 2023; Jain et al. 2013).

In this scenario, the effects of thiazolidine derivatives on peroxisome proliferator-activated receptors (PPARs) are well known (Stark et al. 2021). The PPARs are nuclear receptors that regulate genes involved in metabolism and inflammatory responses. The three primary subtypes are PPARα, PPAR-β/δ, and PPARγ (Jabbari et al. 2021). Upon activation, PPARγ reduces the release of inflammatory cytokines such as TNF-α and IL-6 by inhibiting the nuclear factor kappa B (NF-κB) pathway and activator protein 1 (AP-1). PPARγ also promotes the polarization of macrophages into an anti-inflammatory state (M2 phenotype), which helps resolve the inflammatory process (Chawla et al. 2019).

Some thiazolidine derivatives, such as thiazolidinediones (e.g., pioglitazone and rosiglitazone), function as selective agonists of the PPARγ receptor, directly influencing its activity (Bhattacharyya 2020; Fedchenko et al. 2022). As a negative modulator of excessive and persistent inflammation, the activation of PPARγ facilitates the polarization of macrophages towards an anti-inflammatory phenotype (M2), thereby contributing to the resolution of the inflammatory response (Bassaganya-Riera et al. 2010; Bashir et al. 2016). Macrophages are essential immune cells found in tissues or derived from inflammatory infiltrates. These highly heterogeneous cells exhibit significant plasticity, contributing to both physiological and pathological processes (Funes et al. 2018; Watanabe et al. 2019).

In our previous study (Mohr et al. 2022), several thiazolidine derivatives were tested for their anti-inflammatory activity. After screening 28 compounds, we identified the prominent activity of thiazolidine derivative **13** [methyl 2-(benzoylimino)-3-methyl-4-(4-nitrobenzyl)-1,3-thiazolidine-4-carboxylate]. This compound significantly reduced pro-inflammatory markers in both in vitro (nitric oxide, IL-6, TNF-α, IL-1β, iNOS, COX-2, NF-kB p65) and in vivo models (total leukocyte count, neutrophils, and exudation). Additionally, its immunomodulatory capacity was demonstrated by increasing several anti-inflammatory markers in vitro (IL-4, IL-10, IL-13, Arginase-1, phagocytic index, and CD206). These results underscore the compound ability to repolarize macrophages from the M1 to the M2 phenotype (Mohr et al. 2022). However, no study has been conducted to confirm the potential interaction between thiazolidine **13** and the PPARγ receptors.

Therefore, this study aims to investigate both the PPARγ receptor activation induced by thiazolidine **13** and its effects on macrophage polarization.

## Material and methods

### Synthesis of thiazolidine 13

**Compound 13** [*methyl 2-benzoylimino-3-methyl-4-(4-nitrobenzyl)-1,3-thiazolidine-4-carboxylate*], used in this study, was readily synthesized in the racemic form through a three-step protocol involving the initial preparation of the corresponding isothiouronium salt, followed by *N*-benzoylation with benzoic anhydride and subsequent base-mediated intramolecular anti-Michael addition process, as reported previously (Ferreira & Sá, 2015; Ferreira et al. 2022). This compound, obtained as a racemate (50:50 mixture of each enantiomer *S* and *R*), was purified by crystallization with acetonitrile (or, alternatively, by column chromatography on silica gel) to afford a stable crystalline solid with high purity. **Compound 13** was dissolved in 1% dimethyl sulfoxide (DMSO), following the pre-defined concentrations. After that, the diluted compound was aliquoted and stored at − 80 °C until the moment of the experiments, when the selected compound was properly dissolved in cell culture medium. **Compound 13** was dissolved in phosphate-buffered saline (PBS) and 1% DMSO. The solutions were freshly prepared a few minutes before each experiment.

### RAW 264.7 macrophage cell culture

The RAW 264.7 macrophage cell line was purchased from Rio de Janeiro Cell Bank, Brazil. The cells were maintained in a humidified incubator at 37 °C with 5% CO_2_, and cultured in Dulbecco’s Modified Eagle Medium (DMEM), supplemented with 10% Fetal Bovine Serum (FBS) and 1% antibiotics (100 U/mL penicillin, 100 μg/mL streptomycin). Before the experiments, cell viability was confirmed by the Trypan blue technique. All experiments were performed between the 3rd and 9th passages at 80% confluence. As an adherent line, RAW 264.7 macrophages were washed with PBS and detached using EDTA (Versene®) and cell scrapers. The in vitro assays were performed in triplicate and repeated on different days (*n* = 3/group). All materials were sterilized and experiments were conducted in a contamination-free environment (Mohr et al. 2022).

### LPS-induced RAW 264.7 cell inflammation assay

The experiments were performed using RAW 264.7 macrophages stimulated with LPS (*E. coli*: O111:B4—Sigma Aldrich, 1 μg/mL; or *E. coli*: O26:B6—Sigma Aldrich, 1 μg/mL) (Rossol et al. 2011; Mohr et al. 2022). RAW 264.7 macrophages were seeded in well plates and incubated for 24 h. After incubation, the culture medium was replaced, and the cells were pre-treated for 1 h with the designated concentration of thiazolidine **13** (30 µM), following the predefined group assignments (n = 3/group). The only group not stimulated with LPS was the blank control (A), which received sterile PBS. Following LPS induction, the cells were incubated for an additional 24 h. After this period, supernatants were collected to assess pro- and anti-inflammatory parameters. Our previous study determined the optimal dose of **compound 13** as being 30 μM (Mohr et al. 2022). Therefore, the 30 μM dose was used in all experiments described in this manuscript.

### Measurement of nitric oxide and PPARγ activity investigation

Raw 264.7 cells were stimulated with LPS (*E. coli*: O26:B6—Sigma Aldrich, 1 μg/mL). Macrophages were seeded in 96-well plates (1 × 10^5^ cells/well) and incubated overnight. After incubation, cells were pre-treated (or not) for 1 h with the PPARγ antagonists T0070907 (10 µM, Sigma Aldrich) and GW9662 (Elkahloun et al. 2019; Wen et al. 2018). After the pre-incubation, cells were stimulated with (LPS 1 μg/mL) and treated (or not) with **compound 13** (30 µM) (n = 8/group). Following LPS induction, cells were incubated for an additional 24 h. The supernatant was collected to evaluate nitrite (NO₂⁻), in the supernatant using the Griess reaction (Green et al. 1982). Absorbance was measured at 540 nm using an ELISA plate reader, and NO₂⁻ levels were quantified using a nitrite standard curve (0–200 mM). The results were expressed in µM.

### LPS-induced Raw 264.7 polarization assay

RAW 264.7 cells (1 × 10^5^ cells/well) were seeded in 24-well plates and incubated overnight. Macrophages were either left unstimulated (M0 phenotype) or pre-treated with the PPARγ antagonists T0070907 (10 µM, Sigma-Aldrich) and GW9662 (10 µM, Sigma-Aldrich), followed by stimulation with LPS (*E. coli*: O26:B6—Sigma Aldrich, 1 μg/mL) (M1 phenotype) and treatment with or without **compound 13** (30 µM) for 48 h. Cells were then collected and stained for CD206, CD80 and F4/80 markers (20 min at 4 °C) and evaluated by flow cytometry (C6 BD Accuri™).

### Quantification of pro‑ and anti‑inflammatory cytokines (IL‑6, IL-10, TNF‑α, IL-12p70, IFN-γ and MCP-1) in LPS-induced RAW 264.7 macrophages

RAW 264.7 cells (5 × 10^4^ cells/well) were seeded in 96-well plates and stimulated with LPS (*E. coli*: O111:B4—Sigma Aldrich, 1 μg/mL). After the cell inflammation assay, the supernatant was collected. The levels of pro-inflammatory cytokines (IL-6, TNF-α, IL-12p70, IFN-γ, and MCP-1) and the anti-inflammatory cytokine IL-10 in the supernatant were measured using flow cytometry. This analysis was performed with the BD Bioscience FACVerse™ Flow Cytometer, employing a commercial Cytometric Bead Array (CBA) Mouse Inflammation Kit. The kit uses beads coated with antibodies specific to each cytokine, allowing simultaneous detection of multiple targets. Data were analyzed and quantified using FCAP Array™ software (BD Biosciences, San Jose, CA, USA), and results were expressed as pg/mL, according to the kit’s standards. The experimental groups were: (A) blank control, consisting of non-inflamed cells treated with sterile PBS; (B) negative control, consisting of LPS-stimulated cells pre-treated with vehicle (1% DMSO); (C) positive control, with LPS-stimulated cells pre-treated with Dexamethasone (7 μM), a standard anti-inflammatory drug; and (D) treatment group, composed of LPS-stimulated cells pre-treated with the selected concentration of **compound 13** (30 µM).

### Quantification of TGF-β in LPS-induced RAW 264.7 macrophages

RAW 264.7 cells (5 × 10^4^ cells/well) were seeded in 96-well plates and stimulated with LPS (*E. coli*: O111:B4—Sigma Aldrich, 1 μg/mL). After the cell inflammation assay, the supernatant was used. Cytokine TGF-β was measured using a commercial ELISA kit, following the manufacturer’s instructions. Specific monoclonal antibodies for this cytokine were used, with concentrations determined by interpolation of the standard curve provided with each kit. The BD Biosciences kit (San Diego, CA, USA) was used. Optical densities were measured at 450 nm using an ELISA reader (MB-580; HEALES, Gouwei Road, SZN, China). Results were expressed in pg/mL, following the sensitivity parameters of each assay.

### RT‑qPCR quantification of mRNA expression of FIZZ-1 in LPS-induced RAW 264.7 macrophages

After pre-treatment and LPS (*E. coli*: O111:B4—Sigma Aldrich, 1 μg/mL) induction in 6-well plates (5 × 10^5^ cells/well), RAW 264.7 macrophages were scraped from the plates and centrifuged at 200 g for 5 min at 4 °C to prepare for RNA extraction. RNA was purified using a column-based extraction method with a commercial kit (Zymo Research Corp., Irvine, CA, USA). The concentration of the extracted RNA was measured at 260 nm using a NanoVue® Plus UV–Vis Spectrophotometer (Chicago, IL, USA). The extracted mRNA was then reverse-transcribed into complementary DNA (cDNA) for quantitative PCR (RT-qPCR) analysis. The RT-qPCR was performed using specific primers designed to amplify the target genes. GAPDH served as the housekeeping gene, with the following primers: sense 5′-GTG.TCC.GTC.GTG.GAT.CTG.AC-3′ and antisense 5′-GGA.GAC.AAC.CTG.GTC.CTC.AG-3′. For the FIZZ-1 gene, the primers were: sense 5′-CCA.ATC.CAG.CTA.ACT.ATC.CCT.AC-3, and antisense 5′-ACC.CAG.CAG.CAG.TCA.TCC.CA-3′. The RT-qPCR reactions were performed in triplicate, and amplification was carried out using the following thermocycler conditions: 5 min at 95 °C, followed by 40 cycles of 45 s at 95 °C, 45 s at 58 °C, and a final step of 45 s at 72 °C. Gene expression was quantified using the 2^⁻ΔΔCT^ method, with the results compared to the control group (S) for relative expression analysis.

### THP-1 macrophage culture

The THP-1 macrophage cell line was purchased from the Rio de Janeiro Cell Bank, Brazil. THP-1 cells were cultured in RPMI-1640 medium (ATCC 30-2001), supplemented with 10% fetal bovine serum (FBS) and 2-mercaptoethanol at a final concentration of 0.05 mM. Due to its limited stability, 2-mercaptoethanol was added immediately before seeding at a ratio of 0.9 µL per mL of medium. Cultures were maintained at 37 °C in a 5% CO₂ incubator, with complete medium exchanges performed weekly by centrifugation and resuspension in fresh medium. To induce differentiation into macrophage-like cells, THP-1 cells were treated with 200 nM of 12-myristate 13-acetate (PMA) for three days, during which they adhered to the substrate and acquired a macrophage-like morphology.

### LPS-induced THP-1 cell inflammation assay

After differentiation into macrophages, the THP-1 cell line was stimulated with LPS (E. coli: O111:B4—Sigma Aldrich) at a concentration of 2 μg/mL. Briefly, THP-1 macrophages were seeded in 96-well plates (5 × 10^4^ cells/well) and incubated for 24 h. After incubation, the culture medium was replaced, and the cells were pre-treated for 1 h with the designated concentrations of **compound 13**, following the predefined group assignments (n = 3/group).

### Cell viability (cytotoxicity) in LPS-induced THP-1 macrophages

The cytotoxicity of **compound 13** was assessed using the Resazurin (Alamar Blue®) assay, as previously described (O’Brien et al. 2000). THP-1 cells (5 × 10^4^ cells/well) were cultured in 96-well plates for 24 h, then treated with compound 13 at concentrations ranging from 1 to 100 μM for an additional 24 h. After treatment, the medium was replaced with resazurin solution (1.5 mg/mL diluted 1:10 in culture medium) and incubated for 2 h. Cell viability was measured by fluorescence at 530/590 nm, with the vehicle group (1% DMSO) as control, and blank cell viability set to 100%. The CC_10_ value, representing the concentration that kills 10% of cells, was calculated using nonlinear regression analysis via Prism 8.0 software (GraphPad).

### Measurement of nitric oxide in LPS-induced THP-1 macrophages

Nitric oxide (NO) production was assessed by measuring its metabolite, nitrite (NO₂⁻) in the supernatant using the Griess reaction (Green et al. 1982). After treatment with **compound 13** and stimulation with LPS (2 μg/mL), supernatant samples were mixed with an equal volume of Griess reagent and incubated for 40 min at room temperature. Absorbance was measured at 540 nm using an ELISA plate reader, and NO₂⁻ levels were quantified with reference to a nitrite standard curve (0–100 μM). The experiments were conducted in two stages. In the first stage, a NO concentration–response curve (1–100 μM) was performed to confirm the activity of **compound 13** in THP-1 macrophages. In the second stage, the optimal concentration of the compound was used to compare the effects of **compound 13** with the reference drug pioglitazone. The experimental groups were as follows: Blank (B): cells treated only with the vehicles (DMSO and PBS); LPS: cells stimulated with LPS (2 μg/mL); Dexa: cells treated with dexamethasone (7 μM) and stimulated with LPS (2 μg/mL); Pio: cells treated with pioglitazone (3 μM) and subsequently stimulated with LPS; Pio + 30: cells treated with pioglitazone (3 μM) and **compound 13** (30 μM); 30: cells treated with **compound 13** (30 μM) and subsequently stimulated with LPS (2 μg/mL).

### HeLa cell culture

The HeLa cell line was obtained from Rio de Janeiro Cell Bank, Brazil. Cells were cultured in 75 cm^2^ culture flasks (Kasvi, São José dos Pinhais, PR, Brazil) containing DMEM or high-glucose DMEM supplemented with 10% fetal bovine serum (FBS) and 1% antibiotics (100 U/mL penicillin and 100 μg/mL streptomycin), and incubated at 37 °C (humidified atmosphere of 5% CO₂). Once cultures reached over 80% confluence, cells were subcultured for the experiments (Benvenutti et al. 2024).

### Cell transactivation assay and reporter gene

To investigate the effects of **compound 13** on the transcriptional activity of PPAR isoforms, HeLa cells (25 × 10^3^ cells per well in a 48-well plate) were co-transfected with plasmids encoding chimeric nuclear receptors composed of the ligand-binding domain of PPARα, PPARβ/δ, or PPARγ fused to the DNA-binding domain of the yeast GAL4 transcription factor (Benvenutti et al. 2024). Briefly, cells were transfected with a plasmid containing a GAL4-responsive element linked to a luciferase reporter gene. Transfections were carried out using Lipofectamine 2000 (Invitrogen) according to the manufacturer’s protocol. After transfection, cells were incubated at 37 °C in a 5% CO₂ atmosphere and treated with vehicle (DMSO), rosiglitazone (PPARγ agonist, 10 µM), pioglitazone (PPARγ agonist, 10 µM), GQ-16 (PPARγ agonist, 10 µM) or increasing concentrations of MF (ranging from 0.1 to 100 µM) for 24 h. Luciferase activity was then measured using the Reporter Luciferase Assay Kit (Promega) according to the manufacturer’s instructions, with luminescence measured on a GloMax 20/20 luminometer (Promega). Results were expressed as fold induction relative to vehicle-treated controls.

### Statistical analysis

The data were analyzed using GraphPad Prism® version 8.0 (San Diego, CA, USA). Results were expressed as the mean ± standard error of the mean (S.E.M.) for parametric data. Residuals were tested for normality with the Shapiro–Wilk test and for homoscedasticity with the Bartlett test. Since the data met both assumptions, one-way ANOVA followed by Tukey’s post hoc test was performed. Statistical significance was set at P < 0.05.

### Docking studies

Docking studies were performed to elucidate the binding modes of the (*R*) and (*S*) enantiomers of **compound 13** at the PPARγ receptor. Pioglitazone, as well as both (*R*) and (*S*) enantiomers, were considered as a modern reference ligand and a well-known total agonist. All ligand structures were optimized using the MMFF94s force field implemented in Avogadro (version 1.2.0), applying the software’s default convergence settings (Hanwell et al. 2012). Docking analyses were performed using the PPARγ protein structure (PDB ID: 2PRG), obtained from the RCSB Protein Data Bank. This structure includes a co-crystallized inhibitor (BRL—rosiglitazone), which was used as a reference to define the binding site. The search space was centered on the coordinates of the BRL ligand (center_x = 50.345; center_y = − 38.214; center_z = 19.575, all in Å), with dimensions of 36.0 Å along each axis (X, Y, Z). The docking calculations were carried out using the AutoDockTools and AutoDock Vina (version 1.1.2) programs, with default settings applied to all parameters, except for exhaustiveness, which was increased to 64 to enhance the accuracy of the docking results (Morris et al. 2009). The residues CYS 285, ARG288, SER289, HIS323, LEU330, ILE341, PHE 363, MET 634, and TYR 473 were treated as flexible during the calculations, in order to consider, the main effects of the induced fit. Molecular interactions identified from the docking results were analyzed using the BINANA software (Durrant and McCammon 2011), with default parameters except for the hydrogen-bond distance, which was set to 3.5 Å to refine interaction detection. Finally, PyMOL (pymol.org) was chosen for molecular visualizations.

## Results

### Measurement of nitric oxide and PPARγ activity investigation

**Fig. 1:**
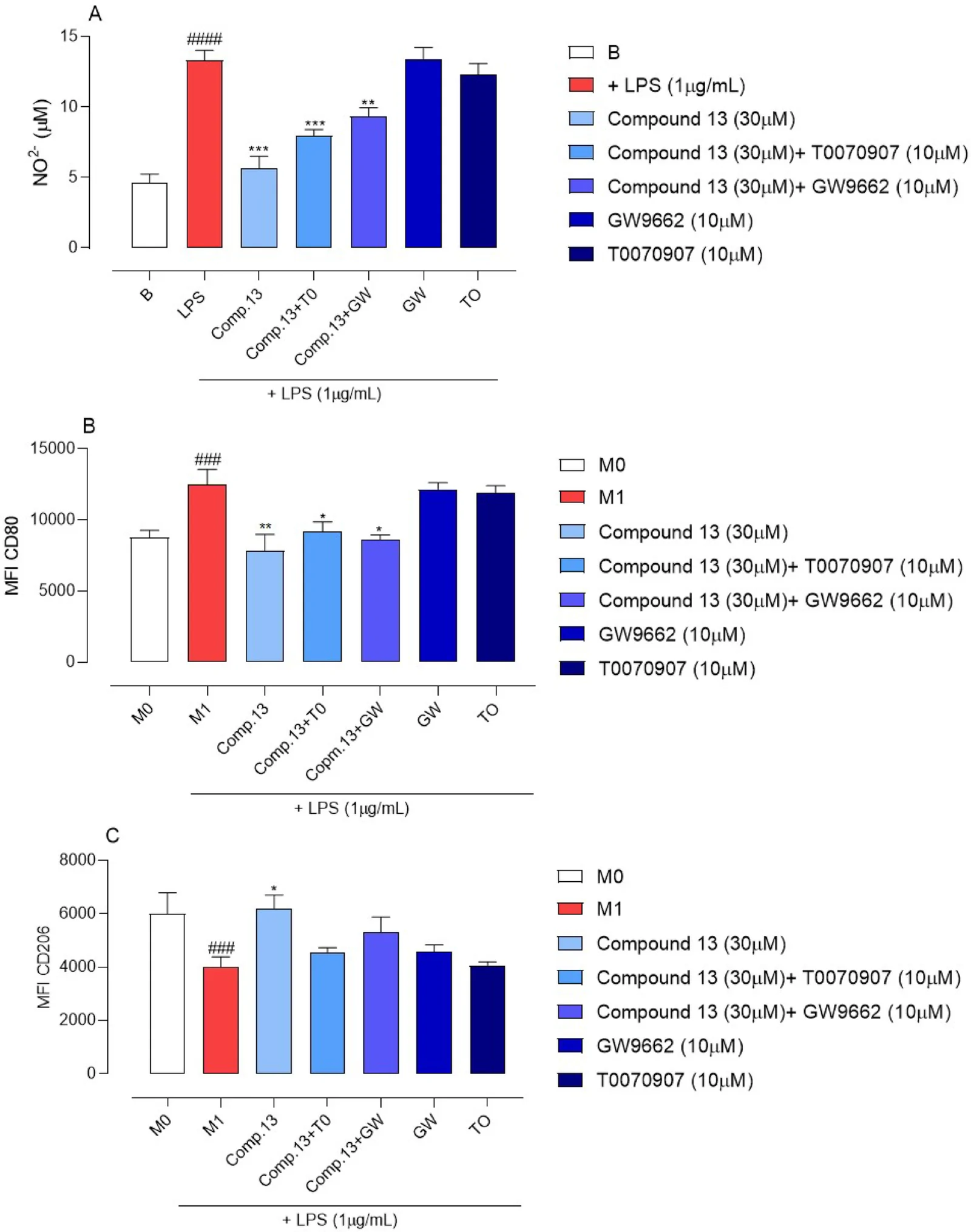
Measurement of Nitric Oxide, PPARγ activity investigation and macrophage polarizationª. ªThe activity of **compound 13** on the PPARγ pathway was determined by measuring NO₂⁻ levels using the Griess reaction (**A**) (*P* < 0.001). The expression of CD80 (**B**) (*P* < 0.01) and CD206 (**C**) (*P* < 0.05) was determined by flow cytometry (C6 BD Accuri). Supernatants were used to quantify NO₂⁻, CD80, and CD206 separately. B: cells pre-treated with vehicle and stimulated with PBS; LPS: cells pre-treated with vehicle and stimulated with LPS (1 μg/mL); **Comp. 13:** cells pre-treated with **compound 13** (30 μM) and stimulated with LPS (1 μg/mL); **Comp. 13** + T0: cells pre-treated with **compound 13** (30 μM) combined with the PPARγ antagonist T0070907 (10 μM) and stimulated with LPS (1 μg/mL); **Comp. 13** + GW: cells pre-treated with **compound 13** (30 μM) combined with GW9662 (10 μM) and stimulated with LPS (1 μg/mL); GW: cells pre-treated only with GW9662 (10 μM) and stimulated with LPS (1 μg/mL); T0: cells pre-treated with T0070907 (10 μM) and stimulated with LPS (1 μg/mL). For flow cytometry experiments: M0, macrophages not stimulated with LPS; M1, macrophages stimulated with LPS (1 μg/mL). The experiments were performed in triplicate, and results were expressed as the mean ± S.E.M. of absolute values; All results were compared with the LPS-inflamed control (####), *P* < 0.05, *P < 0.01, and *P* < 0.001

As expected, **compound 13** was able to reduce NO₂⁻ release in LPS-stimulated RAW 264.7 macrophages. Treatment with **compound 13** (30 µM) alone significantly reduced NO₂⁻ levels (% of inhibition: 57.7 ± 17.2) (*P* < 0.001) (Fig. 1A). Although smaller, the effect was partially maintained in the presence of the PPARγ antagonists T0070907 (10 µM) (% of inhibition: 30.1 ± 12.7; *P* < 0.01) and GW9662 (10 µM) (% of inhibition: 40.1 ± 8.2; *P* < 0.001) (Fig. 1A), suggesting a possible partial independence from the PPARγ pathway. When administered alone, the antagonists GW9662 and T0070907 did not reduce NO₂⁻ production (Fig. 1A).

### LPS-induced Raw 264.7 polarization assay (CD80-M1 and CD206-M2)

The analysis of CD80 expression revealed that treatment with **compound 13** significantly reduced the CD80 expression compared to the M1 group (% of inhibition: 37.2 ± 15.8) (*P* < 0.01) (Fig. 1B). This effect was also observed in groups treated with **compound 13** in combination with the PPARγ antagonists T0070907 (% of inhibition: 30.7 ± 3.9; *P* < 0.05) and GW9662 (% of inhibition: 26.7 ± 9.6; *P* < 0.05) (Fig. 1B), reinforcing the hypothesis of a modulation that does not rely exclusively on the PPARγ pathway.

The analysis of the CD206 expression, characteristic of M2 polarization, demonstrated that **compound 13** significantly increased the expression of this marker in LPS-stimulated RAW 264.7 macrophages only in the group treated exclusively with **compound 13** (% of increase: 53.6 ± 22.6; *P* < 0.05) (Fig. 1C). The increase in CD206 expression was not observed in the presence of the antagonists T0070907 and GW9662 (Fig. 1C).

### Quantification of pro‑ and anti‑inflammatory cytokines in LPS-induced RAW 264.7 macrophages

**Fig. 2:**
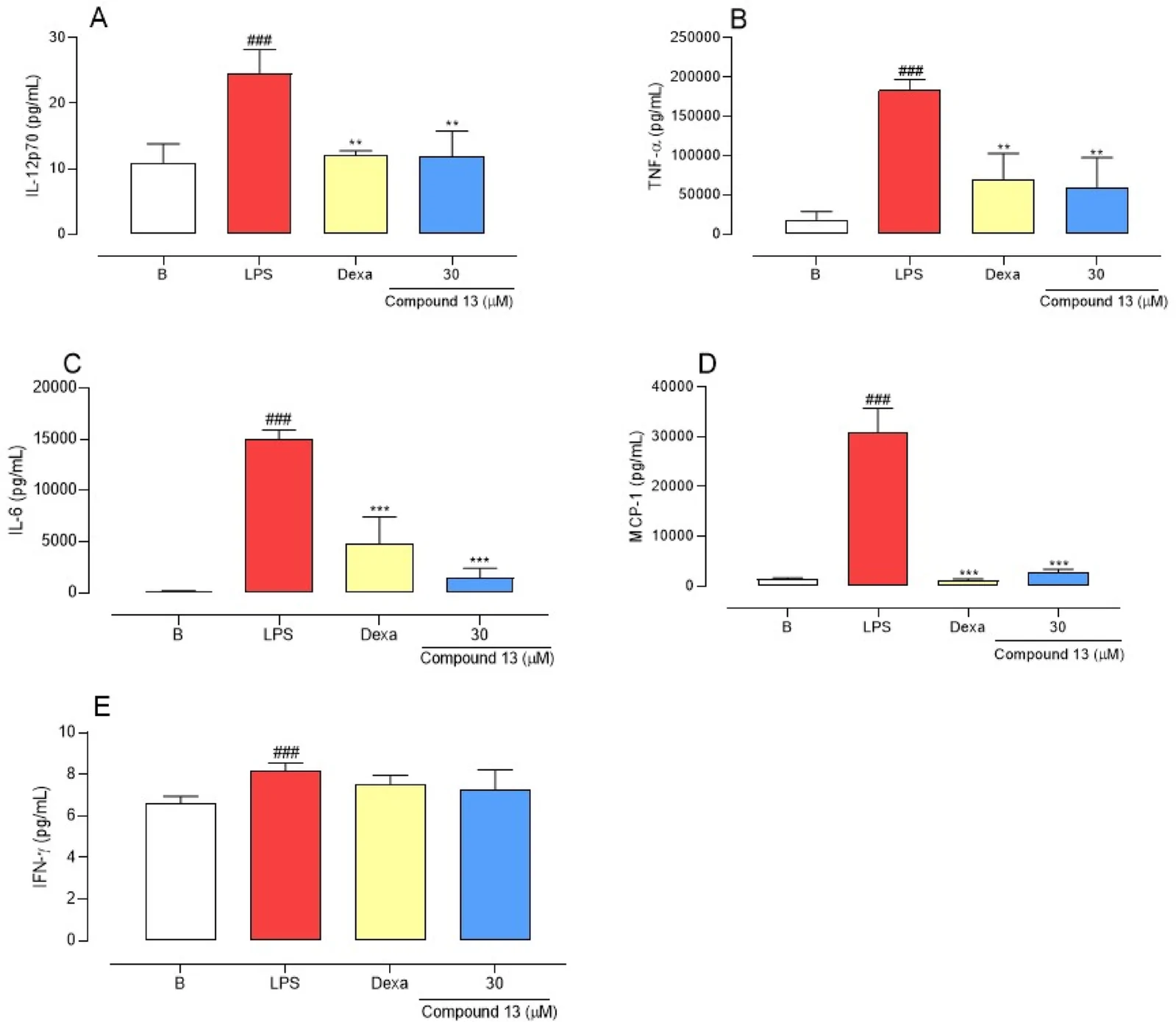
Quantification of pro-inflammatory cytokines in LPS-induced RAW 64.7 macrophagesª. ªEffect of **compound 13** at 30 μM dose on IL-12p70 (**A**), TNF-α (**B**), IL-6 (**C**), MCP-1 (**D**), and IFN-γ secretion (**E**) in LPS-induced RAW 264.7 macrophages. B: cells pretreated with vehicle and stimulated with PBS; LPS: cells stimulated with LPS (1 μg/mL); Dexa: cells pretreated with dexamethasone (7 μM) and stimulated with LPS (1 μg/mL); **Compound 13**: cells pre-treated with 30 μM of **compound 13** and stimulated with LPS (1 μg/mL). The experiments were performed in triplicate and the results were expressed as the mean ± S.E.M. of the absolute values; All results were compared with the LPS-inflamed control (####); *P < .0.05, **P < 0.01, and ***P < 0.001

**Compound 13** demonstrated the capacity to significantly suppress the production of pro-inflammatory cytokines, including IL-12p70 (% inhibition: 42.6 ± 6.19.2; *P* < 0.05) (Fig. 2A), TNF-α (% inhibition: 56.2 ± 1.4; *P* < 0.05) (Fig. 2B), IL-6 (% inhibition: 90.4 ± 6.3; *P* < 0.001) (Fig. 2C), and the chemokine MCP-1 (% inhibition: 90.6 ± 1.7; *P* < 0.001) (Fig. 2D). Given that these biomarkers are indicative of inflammation, the positive control, dexamethasone, also exerted a suppressive effect on the release of IL-12p70 (% inhibition: 44.4 ± 6.9; *P* < 0.05) (Fig. 2A), TNF-α (% inhibition: 61.5 ± 17.8; *P* < 0.05) (Fig. 2B), IL-6 (% inhibition: 67.8 ± 16.9; *P* < 0.001) (Fig. 2C), and MCP-1 (% inhibition: 96.4 ± 1.2; *P* < 0.001) (Fig. 2D). Notably, IFN-γ remained unchanged in the LPS control group across both treatment conditions, with dexamethasone and **compound 13** (Fig. 2E).

**Fig. 3:**
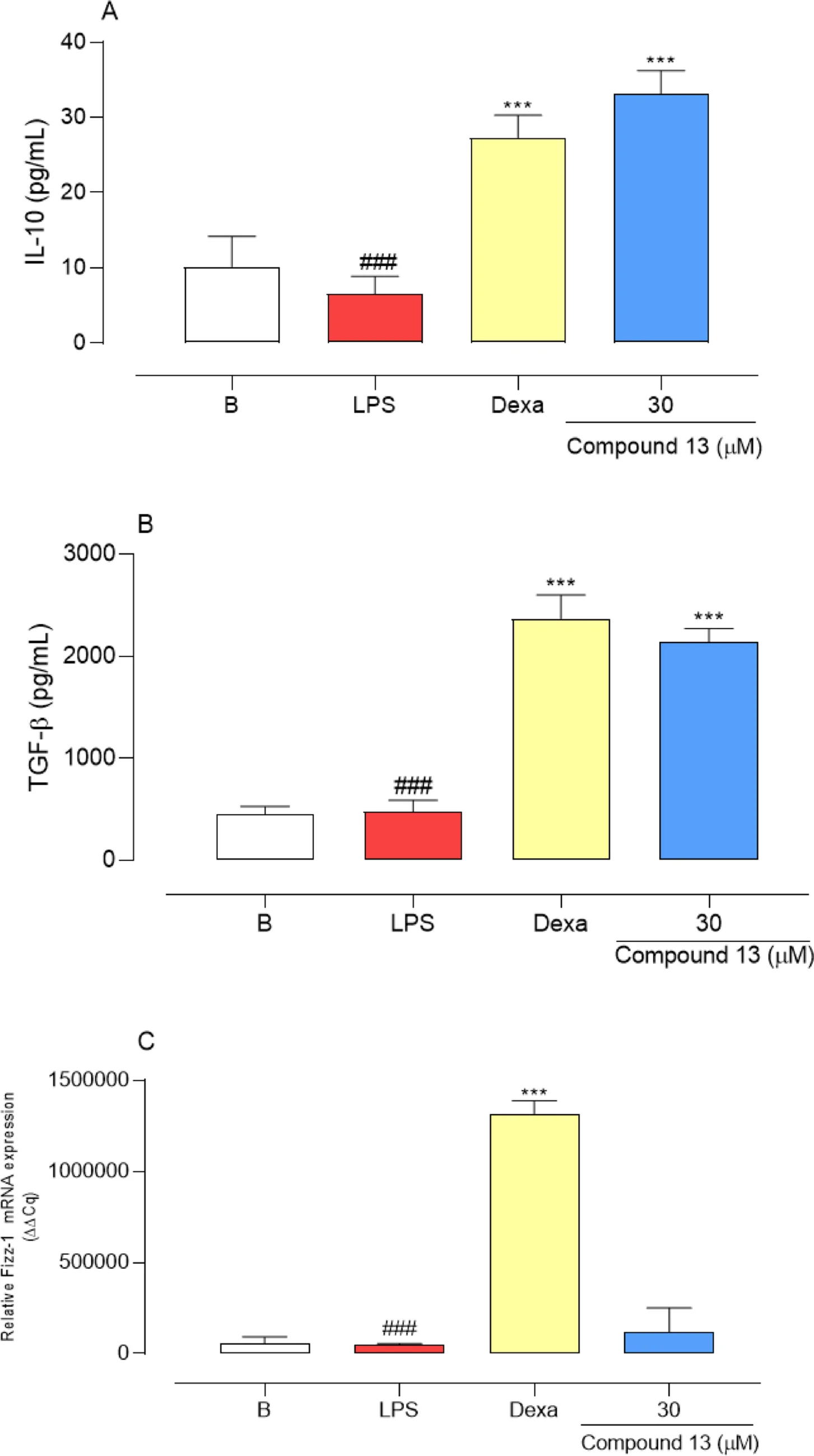
Quantification of anti-inflammatory cytokines and FIZZ-1 expression using LPS-induced RAW 264.7 macrophagesª. ªEvaluation of the effect of **compound 13** on IL-10 (**A**), TGF-β (**B**) and FIZZ-1 (**C**) expression in RAW 264.7 macrophages stimulated with LPS. B: cells pre-treated with vehicle and stimulated with PBS; LPS: cells pre-treated with vehicle and stimulated with LPS (1 μg/mL); Dexa: cells pre-treated with dexamethasone (7 μM) and stimulated with LPS (1 μg/mL); **Compound 13**: cells pre-treated with **compound 13** (30 μM) and stimulated with LPS (1 μg/mL). IL-10 was measured by flow cytometry, and TGF-β was evaluated by ELISA. FIZZ-1 mRNA was calculated in duplicate by the relative CT method in comparison to the (B) group results (2-ΔΔCT). The results were expressed as the mean ± S.E.M.; n = 3; All results were compared with the LPS-inflamed control (####), **P < 0.01 and ***P < 0.001. ###*P* < 0.001

Concomitantly, **compound 13** promoted a significant enhancement in the release of the anti-inflammatory cytokines IL-10 (% increase: 329.1 ± 40.5; *P* < 0.001) (Fig. 3A) and TGF-β (% increase: 349.4 ± 28.2; *P* < 0.001) (Fig. 3B). Similarly, dexamethasone elicited an increase in IL-10 secretion (% increase: 253.9 ± 38.7; *P* < 0.001) (Fig. 3A) and TGF-β secretion (% increase: 398.2 ± 48.4; *P* < 0.001) (Fig. 3B).

### RT‑qPCR quantification of mRNA expression of FIZZ-1 in LPS-induced RAW 264.7 macrophages

**Compound 13** did not exhibit the ability to enhance FIZZ-1 expression in LPS-stimulated RAW 264.7 macrophages, in contrast to the reference drug dexamethasone, which significantly upregulated the expression of this transcription factor (% increase in FIZZ-1: 2669.4 ± 163.2) (Fig. 3C).

### Cell viability (cytotoxicity) in LPS-induced THP-1 macrophages

**Compound 13** did not show cytotoxicity toward the THP-1 cell line, with a CC_10_ above the highest concentration tested (CC_10_ > 100 μM) (Supplementary material, Figure S1A).

### Measurement of nitric oxide in LPS-induced THP-1 macrophages

As initially described in the Materials and Methods section, NO production was measured to confirm the anti-inflammatory activity of **compound 13** in LPS-stimulated THP-1 macrophages. In these experiments, **Compound 13** significantly reduced NO release at the two highest concentrations tested (30 and 100 μM) (% inhibition: 59.0 ± 15.5 and 64.7 ± 7.6; *P* < 0.01) (Supplementary Material, Figure S1B). As expected, the reference drug dexamethasone also reduced NO production (% inhibition: 92.3 ± 4.6; *P* < 0.001). No significant difference was observed between the 30 and 100 μM concentrations (t-test). Therefore, the 30 μM concentration of **compound 13** was selected for subsequent experiments (Supplementary Material, Figure S1B).

The activity of **compound 13** (30 μM) was then compared with the reference drug, pioglitazone. **Compound 13** reduced NO production in both evaluated groups (Pio + 30 and 30) (% inhibition: 44.3 ± 11.4 and 60.0 ± 15.1; *P* < 0.001) (Supplementary Material, Figure S1C). As expected, dexamethasone and pioglitazone also decreased NO release (% inhibition: 92.3 ± 4.0 and 44.0 ± 11.4; *P* < 0.001 and *P* < 0.05, respectively) (Supplementary Material, Figure S1C).

### Cell transactivation assay and reporter gene

**Fig. 4:**
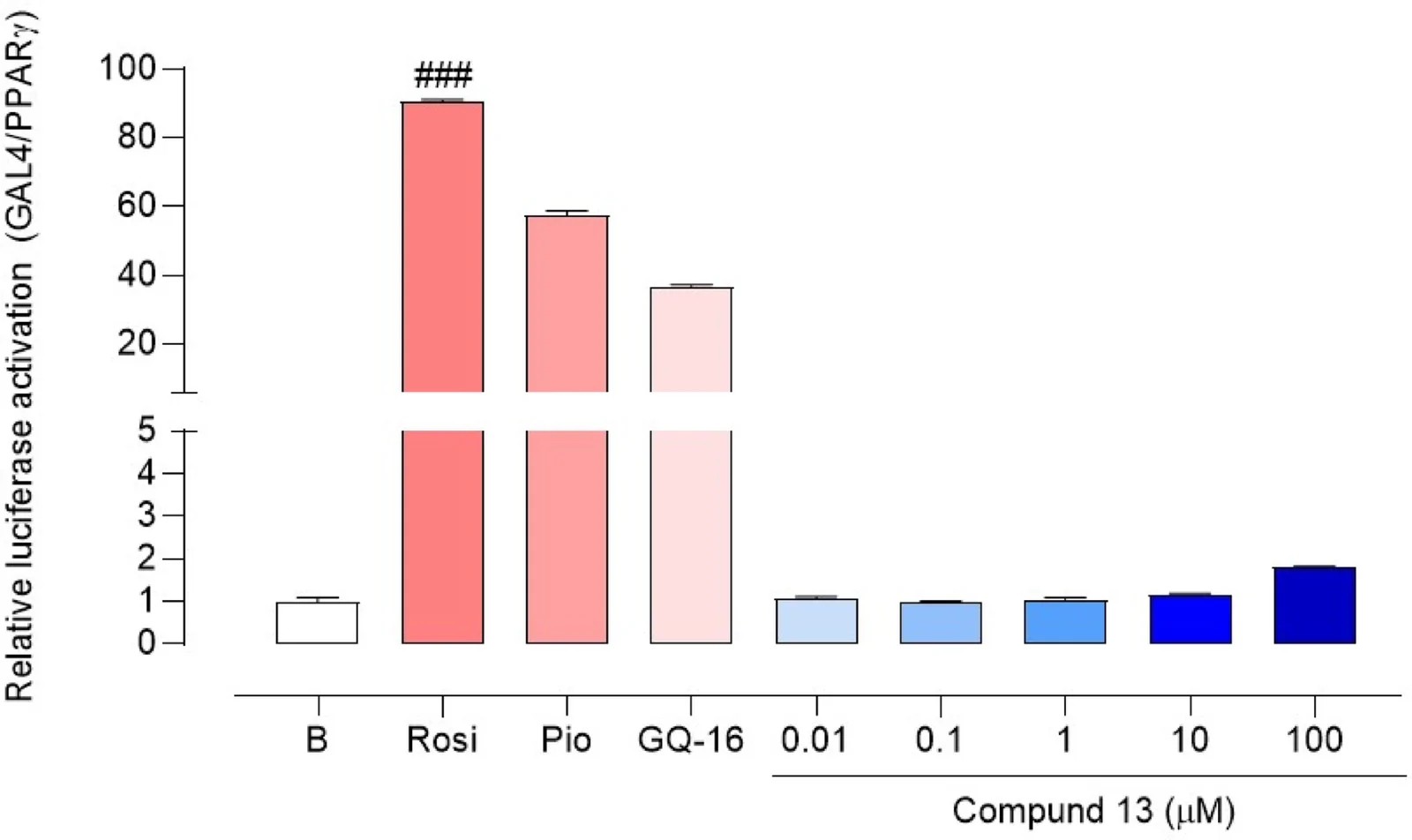
Cell transactivation assay and reporter gene using HeLa cellsª. ªEvaluation of **compound 13** effects on PPARγ nuclear receptor transcriptional activity using a HeLa cell-based transactivation assay with a reporter gene system. B: cells pretreated with vehicle and stimulated with PBS; Rosi: cells treated with Rosiglitazone (PPARγ agonist, 10 µM); Pio: cells treated with Pioglitazone (PPARγ agonist, 10 µM); GQ-16: cells treated with GQ-16 (PPARγ agonist, 10 µM); **Compound 13**: cells pre-treated with **compound 13** (0.01 to 100 µM). The experiments were performed in triplicate and the results were expressed as the mean ± S.E.M. of the absolute values; the positive control Rosi (###) was used as a comparative; *P < .0.05, **P < 0.01, and ***P < 0.001

The final step involved assessing the effects of **compound 13** on the transcriptional activity of the nuclear receptor PPARγ using a transactivation assay in HeLa cells and a reporter gene system. As shown in Fig. 4, PPARγ isoforms were effectively activated by their respective agonists—rosiglitazone, pioglitazone, and GQ-16. In contrast, **compound 13** did not significantly affect PPARγ activation, inducing less than 5% of the transcriptional activity observed with the full agonist rosiglitazone (Fig. 4). Notably, **compound 13** showed no cytotoxicity against HeLa cells at any concentration tested in this assay (data not shown). All previous results (Mohr et al. 2022) and the additional markers are presented in Table 1.

**Table 1:** Main characteristic of M1/M2 phenotype macrophages

Phenotype	Suggested roles	Related cytokines	Other markers	Main compound 13’ biomarkers
M1	Pro-inflammatory activity	IFN-γ: IFN-γ, IL-1β, IL-6, IL-8 LPS: IL-10 low, IL-12, IL-18, IL-23, TNF-α	CXCL9, CXCL10, CXCL11, Arginase II, iNOS, LPS	IFN-γ
M2a	Allergy, probibrotic, anti-inflammatory, wound healing	IL-4, L-10, TGFβ1, IL-1RA, IL-13, IL-12 low, IL-23 low	Arginase I, FIZZ1/RELMα (mouse), Ym1 (mouse), YM2, estabilin-1, CD163, CD206, PPARγ	IL-4, L-10, IL-12low, TGFβ1, Arginase-I, CD206, PPARγ
M2b	Th2 activation, imune regulation, promoting infection, tumor progression	IL-1β, IL-6, IL-10, TNF-α	CD86, SPHK1/2	IL-10
M2c	Immunosuppression, phagocytosis, tissue repair, matrix remodelation	IL-10, IL-6, TGF-β	Arginase I, CXCL13, CD163, CD206, CXCR4, MerTK	IL-10, TGF-β, Arginase-I, CD206, phagocytosis
M2d	Tumor progression, angiogenesis, clearance of apoptotic tissue	IL-10, IL-12 low, TNF-α low, TGF-β	VEGF, iNOS (mouse)	IL-10, IL-12 low, TNF-α low, TGF-β

The table summarizes the main macrophage phenotypes, classically activated (M1) and alternatively activated (M2a, M2b, M2c, and M2d), described in the literature. Several studies were considered to list the suggested roles, the main cytokines expressed in each phenotype, and other markers most frequently associated with each macrophage phenotype. The main biomarkers evaluated during the investigation of the mechanism of action and anti-inflammatory activity of **compound 13** were described by our research group. Additional information was adapted from Chinetti-Gbaguidi et al. 2015; Gharavi et al. 2022; Strizova et al. 2023

### Docking studies

**Fig. 5:**
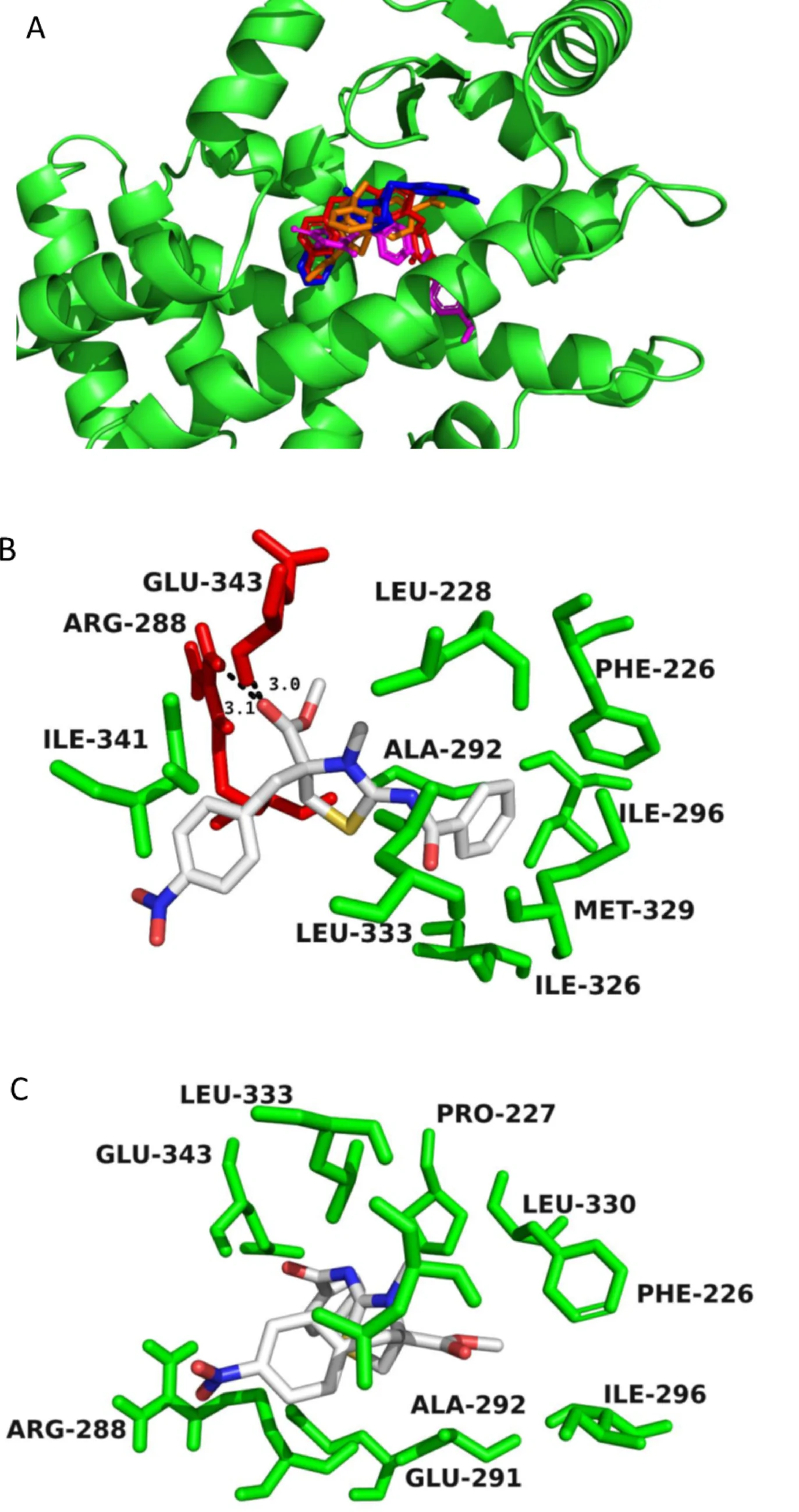
Docking results of compound 13 at PPARγ binding siteª. ªDocking results of compound 13 at PPARγ binding site Docking results at the PPARγ binding site. **A** PPARγ (green) binding site with (S)-compound 13 (blue), (R)-compound 13 (orange), (R)-( +)-pioglitazone (red), and (S)-(-)-pioglitazone (magenta). **B** Interaction profile of (S)-compound 13. PPARγ residues involved in hydrophobic interactions (green) and hydrogen bonds (red) are shown. **C** Interaction profile of (R)-compound 13. PPARγ residues involved in hydrophobic interactions (green) are shown. Colors in panels B and C represent interaction types and do not correspond to ligand identity

A comprehensive docking analysis was performed to elucidate the individual interactions of each (*S*) and (*R*) enantiomer of **compound 13** with PPARγ, in comparison to the (*S*)-(-) and (*R*)-( +) enantiomers of pioglitazone. Figure 5A illustrates the superimposed docking poses of both (*R*) and (*S*) enantiomers of **compound 13**, together with the corresponding enantiomers of pioglitazone, within the PPARγ binding site. Figure 5B shows the binding orientation of the (*S*) enantiomer of **compound 13** and highlights its key molecular interactions with PPARγ. In contrast, Fig. 5C depicts the binding mode and interactions of the (*R*) enantiomer.

As shown in Fig. 5A, all four molecules occupy the same binding pocket within PPARγ. Notably, Fig. 5B reveals that (*S*)-**compound 13** forms key hydrogen bonds with residues ARG288 (3.1 Å) and GLU343 (3.0 Å), indicating strong interactions that may contribute to improving binding stability. Interestingly, Fig. 5C shows that (*R*)-**compound 13** also interacts with ARG288, but via a hydrophobic contact, suggesting a distinct interaction profile that may contribute to its lower binding affinity.

The docking scores for (S)-**compound 13** (− 9.6 kcal/mol), (*R*)-**compound 13** (− 8.7 kcal/mol), (*R*)-( +)-pioglitazone (− 9.4 kcal/mol), and (*S*)-(–)-pioglitazone (− 9.8 kcal/mol) indicate that the (*S*) enantiomer exhibits the highest predicted binding affinity—lowest predicted binding energy—for **compound 13**. The superior predicted affinity of the (*S*) enantiomer of **compound 13** appears to be attributable to key hydrogen bonds between the ligand and critical residues at the PPARγ binding site. As shown in Fig. 5B, (*S*)-**compound 13** also interacts via hydrophobic contacts with residues ALA292, ILE296, ILE326, ILE341, LEU228, LEU333, MET329, and PHE226. Notably, such interactions, particularly with the side chain of ILE341, are known to contribute to the stabilization of the β-sheet region.

**Fig. 6:**
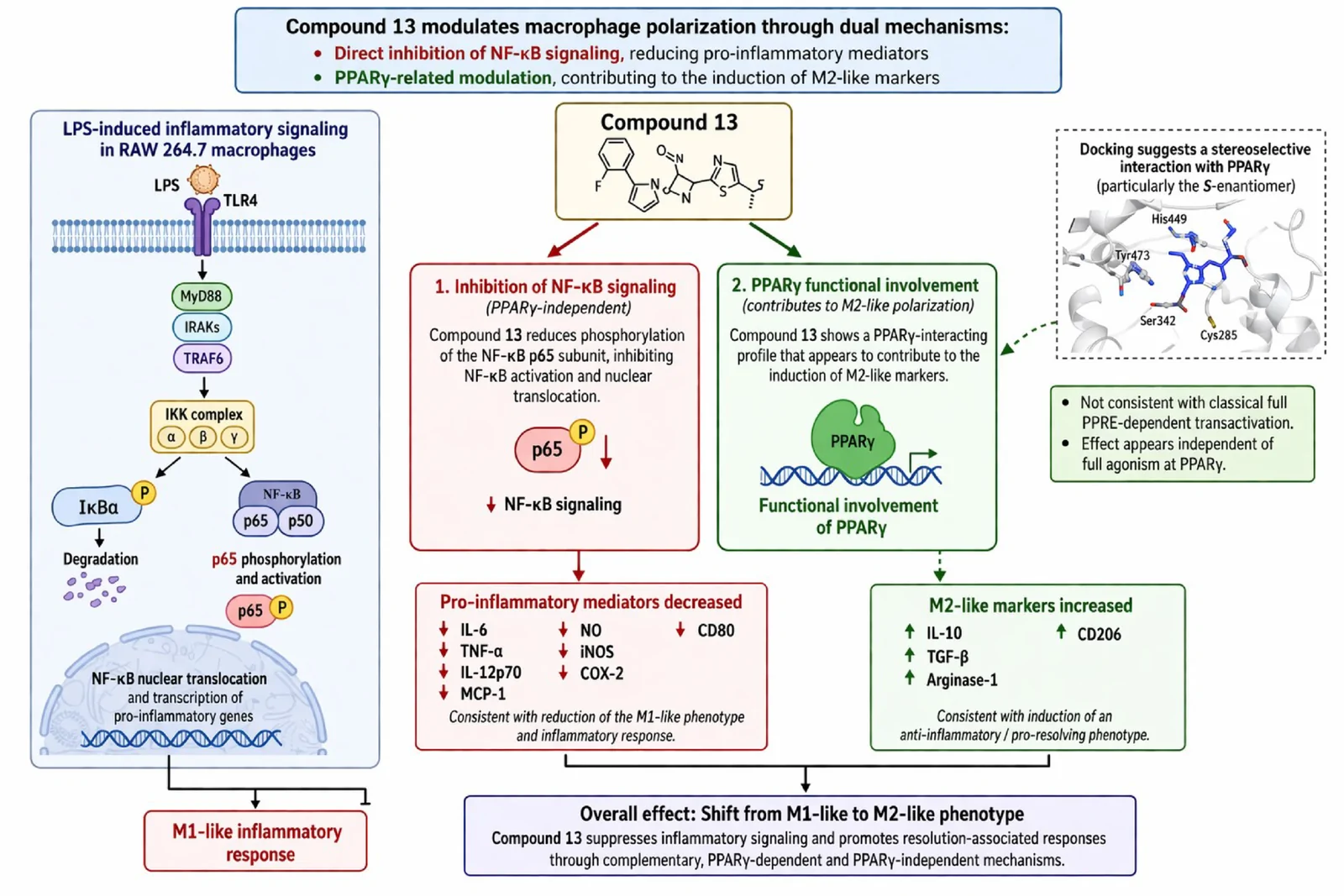
Schematic representation of all the results involving Compound 13 activityª. ªScheme including the signaling pathways involved and other findings related to **compound 13** activity

The binding affinity of (*R*)-**compound 13** is based upon multiple hydrophobic contacts within the PPARγ binding pocket (Fig. 5C). Notably, (*R*)-**compound 13** interacts with several residues also involved in the binding of the (*S*) enantiomer, including ALA292, ILE296, LEU333, and PHE226. In addition, the (*R*) enantiomer makes unique interactions with residues GLU291, GLU343, LEU330, and PRO227. All results, including the signaling pathways involved and other findings, are summarized in Fig. 6.

## Discussion

Our previous study showed that when LPS-induced RAW 264.7 macrophages were pretreated with **compound 13**, nitric oxide production was markedly reduced, suggesting an antagonistic action on the iNOS-mediated inflammatory pathway related to the NF-kB downregulation (Mohr et al. 2022). To confirm the role of PPARγ in this response, NO₂⁻ production was reassessed. During these experiments, the selective antagonists GW9662 and T0070907 were co-administered with **compound 13**. The addition of T0070907 partially reversed the anti-inflammatory activity of **compound 13**, raising NO₂⁻ levels, while GW9662 produced a more modest increase of NO₂⁻. This reversal gradient supports a functional role for PPARγ in the anti-inflammatory activity of **compound 13,** reinforcing that GW9662 and T0070907 (10 µM) do not significantly alter NO production under inflammatory conditions but specifically block the agonistic effect of **compound 13** on PPARγ (Sobolev et al. 2025).

This profile supports a functional involvement of PPARγ in mediating the anti-inflammatory action of **compound 13**, but it should be interpreted with caution when classifying the compound as a partial PPARγ agonist. The incomplete reversal observed in the presence of both antagonists suggests that additional pathways, such as direct modulation of NF-κB or other transcriptional regulators, may also contribute to the observed effects. These findings are consistent with previous reports describing the dual activity of thiazolidine derivatives, which may act through both PPARγ-dependent and -independent mechanisms to regulate macrophage activation and the expression of inflammatory mediators (Lin et al. 2014; Zhou et al. 2021).

This apparent duality can also be observed in the GAL4-PPARγ assay. **Compound 13** did not activate luciferase, even at high concentrations, indicating that it does not behave as a classical or full PPARγ agonist in a PPRE-dependent transactivation system. However, in the NO₂⁻ production assay, the anti-inflammatory effect of **compound 13** proved to be sensitive to PPARγ inhibition. Although these findings may appear contradictory at first glance, they are consistent with the fact that PPARγ exerts functions beyond classical gene transactivation. PPARγ can also act through transrepression of pro-inflammatory factors, such as NF-κB and AP-1, via mechanisms independent of activation of the AF-1 and AF-2 domains responsible for PPRE-driven gene transcription (Hernandez-Quiles et al. 2021). Several studies have shown that selective PPARγ agonists and modulators can elicit anti-inflammatory effects through mechanisms that do not necessarily involve activation of the canonical transactivation pathway (Hou et al. 2012). Moreover, these results corroborate our previous findings showing that **compound 13** exerts its primary anti-inflammatory activity by modulating the NF-κB pathway (Mohr et al. 2022).

Due to the racemic nature of its synthesis, **compound 13** was screened as a racemate (50:50 mixture of the *S* and *R* enantiomers), whereas the docking studies were performed separately for each enantiomer. The docking data suggest that the stereochemistry of **compound 13** plays an important role in its interaction with PPARγ. The (*S*)-enantiomer exhibited a binding mode resembling that reported for known partial agonists, characterized by hydrogen bonding with key residues such as ARG288 and GLU343, together with extensive hydrophobic contacts that stabilize the β-sheet region of the receptor (Nolte et al. 1998). Notably, interactions with ARG288 have been described as a recurrent feature among several PPARγ partial agonists, underscoring the relevance of this residue in ligand binding and receptor modulation (Kroker et al., 2015). Although pioglitazone is clinically administered as a racemic mixture, its enantiomers show no significant pharmacological differences and are known to interconvert in vivo without notable impact (US-FDA, 2017). Nevertheless, docking calculations were performed for both the R and S enantiomers to allow a more detailed comparison with **compound 13**.

The binding interactions predicted for **compound 13** are likely to contribute to ligand–receptor stability and may help explain the higher predicted affinity of the (*S*)-enantiomer relative to its (*R*) counterpart. The (*R*)-enantiomer lacked stabilizing hydrogen bonds and instead relied predominantly on hydrophobic interactions, which may underlie its lower binding affinity. However, these docking results should be interpreted carefully. Although the predicted affinity of the (*S*)-enantiomer was comparable to that of pioglitazone, this does not necessarily imply similar pharmacological behavior, since full and partial agonists may differ not only in affinity, but also in the specific interaction patterns they establish within the ligand-binding pocket and in the conformational changes they induce in the receptor (Kroker et al. 2015). Thus, the stereoselective binding profile of **compound 13** supports the relevance of evaluating individual enantiomers, but does not by itself conclusively demonstrate a partial agonist mechanism.

Therefore, **compound 13** appears to act as a PPARγ-interacting anti-inflammatory modulator, with a profile compatible with partial or selective PPARγ modulation, while exerting anti-inflammatory effects primarily through inhibition of NF-κB signaling. Indeed, PPARγ can inhibit NF-κB activity through transrepression without requiring classical gene transactivation. This process may involve direct interactions with subunits such as p65/p50, leading to suppression of pro-inflammatory genes, including IL-12, in macrophages (Ricote and Glass 2007; Vázquez-Carrera and Wahli 2022). The reduction in CD80 expression observed after treatment with **compound 13** indicates decreased classical pro-inflammatory activation, a feature typically associated with the M1 macrophage phenotype (Raggi et al. 2017). Importantly, this effect persisted even in the presence of PPARγ antagonists, suggesting that modulation of CD80 expression by **compound 13** may involve additional inflammatory pathways beyond PPARγ. In contrast, induction of CD206 expression, a marker of the anti-inflammatory M2-like phenotype, was significantly attenuated by PPARγ antagonists (Croasdell et al. 2015; Rohm et al. 2024). These results suggest that PPARγ contributes more directly to the induction of M2-associated markers in response to **compound 13**, whereas the reduction of pro-inflammatory markers appears to depend more strongly on NF-κB modulation. Overall, the data support a differential regulatory mechanism in which **compound 13** reduces inflammatory activation through both PPARγ-dependent and PPARγ-independent pathways, without supporting an overinterpreted conclusion about a definitive classification as a full or partial agonist.

The results obtained in THP-1 macrophages further support the anti-inflammatory profile of **compound 13**. Importantly, no cytotoxic effects were observed at the tested concentrations, indicating that the reduction in inflammatory mediators was not associated with loss of cell viability. As expected, LPS stimulation significantly increased NO production, confirming the establishment of an inflammatory response. Treatment with **compound 13** significantly reduced NO levels at higher concentrations with no significant difference between these doses, supporting the selection of 30 μM for subsequent experiments. At this concentration, **compound 13** maintained its inhibitory effect on NO production, both when administered alone and in combination with pioglitazone. Notably, the magnitude of inhibition observed for **compound 13** was comparable to that of pioglitazone, while dexamethasone was used as a reference anti-inflammatory drug. These findings reinforce the ability of **compound 13** to attenuate NO production under inflammatory conditions. Although the similar effects observed in the presence of pioglitazone suggest a possible overlap with pathways related to PPARγ modulation, these results should be interpreted with caution and do not support a definitive classification as a classical PPARγ agonist. Instead, they are consistent with a broader anti-inflammatory mechanism, likely involving both PPARγ-related modulation and additional pathways, such as inhibition of NF-κB signaling.

Our previous results demonstrated that **compound 13** reduced phosphorylation of the p65 subunit in LPS-stimulated RAW 264.7 macrophages, thereby inhibiting the NF-κB pathway. This inhibition decreased several pro-inflammatory markers, suppressing iNOS and COX-2 expression and reducing other inflammatory parameters, including NO₂⁻, TNF-α, IL-6, and IL-1β. Simultaneously, **compound 13** significantly increased the release of anti-inflammatory cytokines (IL-4, IL-10, and IL-13), enhancing phagocytic capacity and upregulating CD206 expression on the surface of LPS-stimulated RAW 264.7 macrophages (Mohr et al. 2022). In light of these new results, we hypothesize that the increase in the anti-inflammatory biomarkers evaluated in our previous study is directly attributable to the partial agonism of **compound 13** at the PPARγ signaling pathway.

Thus, treatment with **compound 13** promoted the repolarization of LPS-stimulated RAW 264.7 macrophages from an M1-like state toward an M2-like phenotype. This conclusion is supported by the overall pattern of marker modulation observed after treatment. Although M2 macrophages are currently recognized as a heterogeneous population comprising distinct subtypes with partially overlapping markers and functions, the data from the present study do not allow precise identification of the specific M2 subpopulation induced. Therefore, our results are more appropriately interpreted as evidence that **compound 13** drives macrophages toward an M2-like polarization state. This interpretation is supported by the increased expression of markers commonly associated with alternative macrophage activation, including Arginase-1, CD206, IL-10, and TGF-β, together with the reduction of mediators classically linked to inflammatory macrophage activation, such as IL-12p70, TNFα, IL-1β, IL-6, iNOS, and CD80. Taken together, these findings indicate that **compound 13** promotes a shift in macrophage response toward an anti-inflammatory and pro-resolving profile, consistent with M2-like polarization. The role of alternatively activated macrophages (M2-like) in immunoregulation, resolution of inflammation, tissue remodeling, and repair is well established (Chinetti-Gbaguidi et al. 2015; Martinez and Gordon 2014; Koncz et al. 2023).

An important mechanistic aspect of this response is the activity of **compound 13** on PPARγ. This transcription factor has been widely implicated in macrophage reprogramming toward anti-inflammatory states and in suppressing inflammatory gene expression (Strizova et al. 2023). In macrophages, PPARγ activation by thiazolidinediones reduces the production of pro-inflammatory cytokines such as TNF-α, IL-1β, and IL-6, and downregulates genes including iNOS and MMP9 in a dose-dependent manner (Gharavi et al. 2022). In this context, the ability of **compound 13** to modulate PPARγ provides mechanistic support for both the attenuation of the inflammatory response and the induction of markers associated with M2-like polarization.

The increase in IL-10 and TGF-β, along with the upregulation of Arginase-1 and CD206, further reinforces the interpretation that **compound 13** promotes macrophage functional reprogramming toward a phenotype associated with immunoregulatory and pro-resolving activities. In addition, our previous studies demonstrated increased phagocytic activity in LPS-stimulated RAW 264.7 macrophages following treatment with **compound 13**, which is also compatible with functions frequently attributed to alternatively activated macrophages. However, because these markers are shared across different M2 subsets and are insufficient to discriminate among them, a more specific classification would require investigating additional subtype-specific markers that were not evaluated in the present study (Gharavi et al. 2022). This limitation should be considered in light of the dynamic nature of inflammation, in which macrophage populations are highly plastic and may adopt intermediate or overlapping activation states depending on the local microenvironment (Chinetti-Gbaguidi et al. 2015; Koncz et al. 2023).

The lack of effect of **compound 13** on IFN-γ and FIZZ-1 is also noteworthy. IFN-γ is a canonical mediator of pro-inflammatory macrophage activation, whereas FIZZ-1 is commonly associated with reparative responses and tissue remodeling (Martinez and Gordon 2014; Abdelaziz et al. 2020). The absence of modulation of these markers suggests that **compound 13** does not indiscriminately activate all macrophage-associated programs, but rather promotes a more selective shift toward an anti-inflammatory phenotype. This feature may be particularly advantageous, as it suggests the possibility of controlling inflammatory activation without excessively stimulating pathways that could contribute to unwanted inflammatory or fibrogenic effects. In summary, **compound 13** emerges as a novel partial PPARγ modulator capable of reprogramming macrophage polarization toward an M2-like phenotype. Its effects appear to involve a dual mechanism, combining inhibition of the NF-κB pathway, which contributes to the broad reduction of pro-inflammatory mediators and CD80 expression, with partial PPARγ agonism, which may play a more direct role in the induction of M2-associated markers. The stereochemical analysis further supports the relevance of this mechanism, as the (S) enantiomer of **compound 13** showed a higher predicted binding affinity for PPARγ. Taken together, these findings position **compound 13** as a promising therapeutic candidate for controlling chronic inflammation, acting through integrated PPARγ-dependent and -independent mechanisms to promote macrophage reprogramming toward an M2-like state.

## Supplementary Information

Below is the link to the electronic supplementary material.

Supplementary Material 1.

## Data Availability

This study used previously published data from Mohr et al. (2022), available at https://doi.org/10.1007/s10787-022-01084-x.
