# Correction: The anti‑inflammatory activity of 2‑iminothiazolidines: the role of PPAR-γ and M2 macrophage subpopulations

**DOI:** 10.1007/s10787-026-02422-z

**Published:** 2026-10-12

**Authors:** Eduarda Talita Bramorski Mohr, Tainá Larissa Lubschinski, Pedro Augusto Marocco Moro, Larissa Benvenutti, Natáli Tereza Capistrano Costa, Pedro Luís Pereira Braga de Sousa, Marcus Mandolesi Sá, Marcelo Zaldini Hernandes, Flora Aparecida Milton, José Roberto Santin, Eduardo Monguilhott Dalmarco

**Affiliations:** 1 Department of Clinical Analysis, Center of Health Sciences, Federal University of Santa Catarina (UFSC), Campus Universitario‑Trindade, Florianopolis, SC 88040‑900 Brazil; 2 Department of Chemistry, Federal University of Santa Catarina, Campus Universitario‑Trindade, Florianopolis, SC 88040‑900 Brazil; 3 Postgraduate Program in Pharmaceutical Sciences, Universidade Do Vale Do Itajaí (UNIVALI), Itajaí, SC 88302-901 Brazil; 4 Laboratory of Medicinal Theoretical Chemistry (LQTM), Department of Pharmaceutical Sciences, Federal University of Pernambuco (UFPE), Recife, PE 50740-521 Brazil; 5 Laboratory of Molecular Pharmacology, School of Health Sciences, University of Brasilia (UnB), Brasilia, DF 70910-900 Brazil

**Correction to: Inflammopharmacology (2026) 34:3817–3833** **https://doi.org/10.1007/s10787-026-02253-y**

Regrettably, we have identified technical errors in the chemical structure representation of **Compound 13** in two specific places: the **Graphical Abstract** and **Fig. 6**. These inconsistencies unfortunately went unnoticed during our final proofreading process.

The chemical structure of the tested compound is represented in the wrong way in two places in the manuscript:

1. **Incorrect structure currently published** in the Graphical Abstract (Page 3818).

**Figure:**
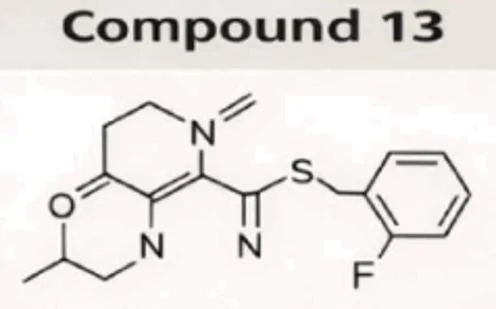

**Correct structure to be replaced**:

**Figure:**
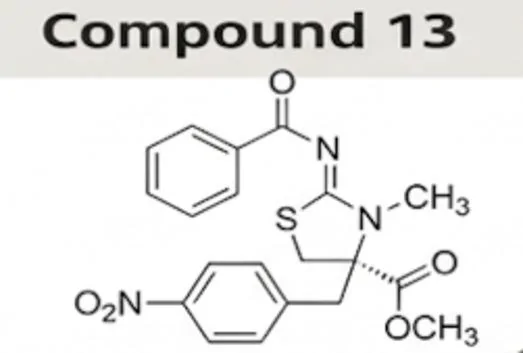

2) **Incorrect structure currently published** in Fig. 6 (Page 3829).

**Figure:**
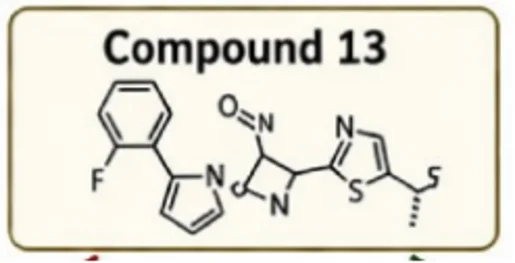

**Correct structure to be replaced**:

**Figure:**
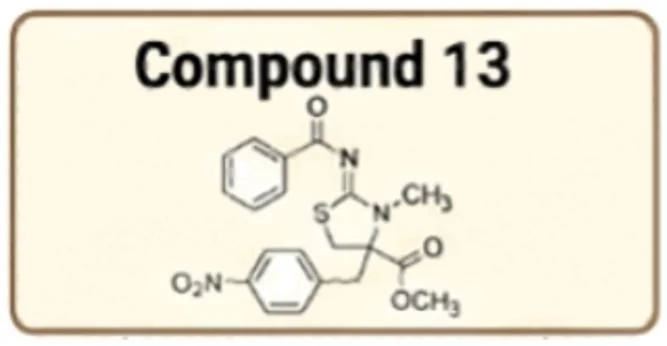

We sincerely apologize for this oversight and for any inconvenience this may cause the editorial team. We are committed to ensuring the highest level of accuracy for the readers of *Inflammopharmacology*.

