# Supplementary material for: The anti‑inflammatory activity of 2‑iminothiazolidines: the role of PPARγ and M2 macrophage subpopulations
Source: Inflammopharmacology. 2026 Apr 22;34(6):3817–33. doi: 10.1007/s10787-026-02253-y (PMC13275556; doi:10.1007/s10787-026-02253-y)

**Supplementary Material**

Figure S1. Anti-inflammatory effects of compound 13 in LPS-stimulated THP-1 macrophagesª.

ª (A) Cell viability of THP-1 macrophages treated with **compound 13** (1–100 μM). No cytotoxic effects were observed (CC₁₀ > 100 μM). (B) Nitric oxide (NO) production in LPS-stimulated THP-1 macrophages treated with increasing concentrations of compound 13 (1–100 μM), using Griess reaction. **Compound 13** significantly reduced NO levels at higher concentrations (30 and 100 μM). (C) NOx production in THP-1 macrophages treated with **compound 13** (30 μM), pioglitazone (3 μM), or their combination (Pio+30), compared with dexamethasone (7 μM) as a reference anti-inflammatory drug. Data are expressed as mean ± S.E.M. All results were compared with the LPS-inflamed control (####).*P < 0.05, **P < 0.01, ***P < 0.001; n.s., not significant.

Figure S1.


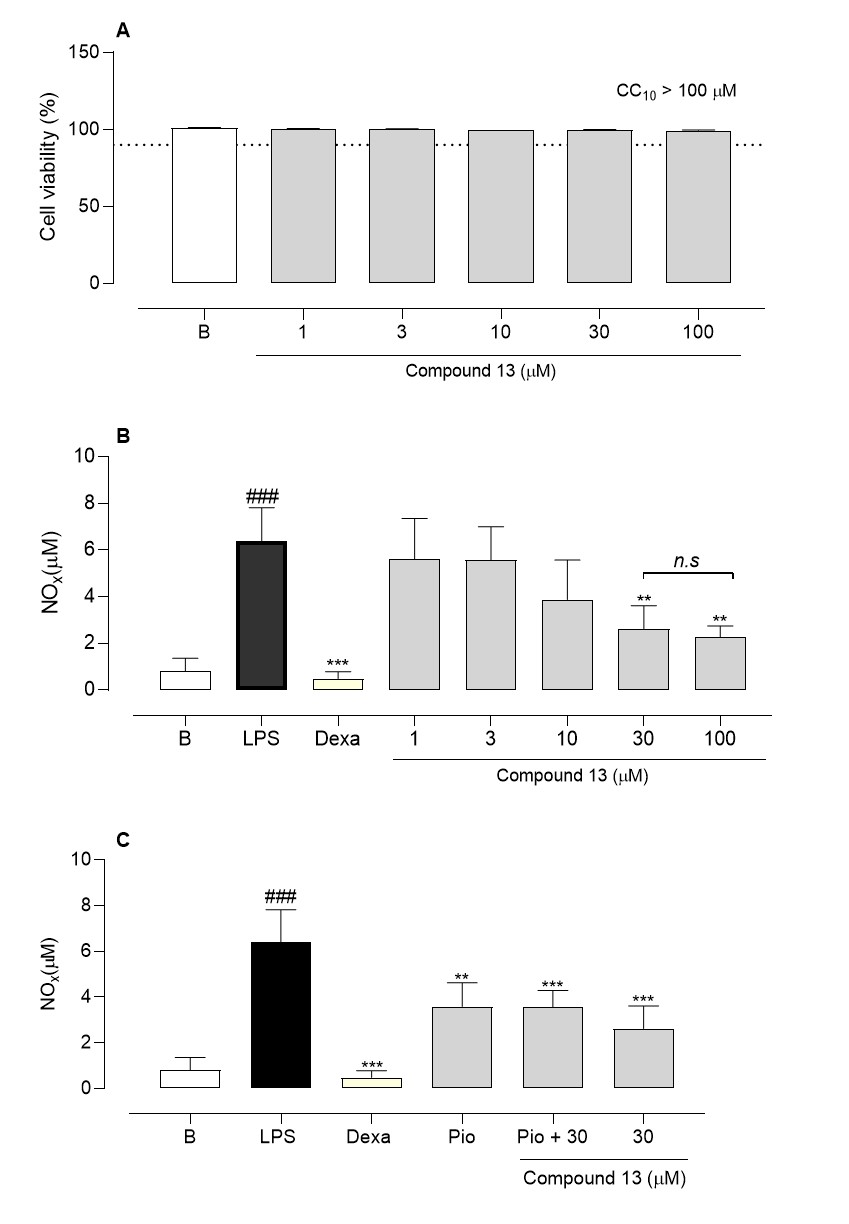

Supplement: Supplementary file 1 — Supplementary Material 1. [file 10787_2026_2253_MOESM1_ESM.docx]
